# Retail food environment in a Brazilian metropolis over the course of a decade: evidence of restricted availability of healthy foods

**DOI:** 10.1017/S1368980022000787

**Published:** 2022-09

**Authors:** Irene Carolina Sousa Justiniano, Mariana Carvalho de Menezes, Larissa Loures Mendes, Milene Cristine Pessoa

**Affiliations:** 1 School of Nutrition, Federal University of Ouro Preto, Rua Dois, Campus Morro do Cruzeiro, CEP, Ouro Preto, Minas Gerais 35.400-000, Brazil; 2 Department of Nutrition, Federal University of Minas Gerais, Avenida Professor Alfredo Balena, Santa Efigênia, Belo Horizonte, Brazil

**Keywords:** Geographic information systems, Food environment, Ultra-processed foods, Food retail, Community food environment, Digital food retail

## Abstract

**Objective:**

To evaluate changes in the retail food environment profile in a Brazilian metropolis over a 10-year period.

**Design:**

An ecological study was conducted in the city of Belo Horizonte, Minas Gerais, Brazil. The addresses of formal food establishments were geocoded and classified according to their sold-food profile. Density changes were analysed according to neighbourhood, population size, income level and geospatial distribution.

**Setting:**

Totally, 468 neighbourhoods in the city of Belo Horizonte, Minas Gerais, Brazil.

**Participants:**

Totally, 83 752 formal food establishments registered for operation in any one or more of those years: 2008, 2011, 2015 and 2018.

**Results:**

There was an increase in unhealthy establishments (154 %), followed by mixed (51 %) and healthy establishments (32 %), during the period evaluated, in addition to an increase in density according to income categories. There was a higher proportion of unhealthy establishments in relation to healthy establishments, indicating worsening of the community food environment over time.

**Conclusions:**

Over the course of 10 years, changes in the neighbourhood’s food environment were unfavourable for adequate access to healthy foods in lower-income neighbourhoods. The findings reinforce the need for interventions aimed at increasing the availability of businesses that offer healthy food in the city.

In recent years, researchers have become increasingly interested in investigating the influence of the food environment on health outcomes. Many have sought to elucidate the relationship between the food environment and obesity, chronic non-communicable diseases, dietary patterns and other health-related factors^(1–5)^. Widely explored aspects of the food environment include the distribution, type and access to commercial food establishments as well as the price, quality and variety of available food^(6–8)^.

The environment in which individuals live has undergone numerous changes in recent years, highlighting those related to the nutritional transition, whose main features are reduced physical activity, changing aspects of eating and dietary patterns^(9)^. In most countries, there has been a significant increase in the availability of foods, especially ultra-processed foods, which are often offered in larger portions and at low prices^(7–10)^. The consumption of these foods is associated with several non-communicable diseases and overweight status^(11–13)^.

Among the various factors associated with food choices, the unequal availability of food outlets in different regions of cities is known to potentially influence the food consumption of individuals, particularly in socio-economically vulnerable neighbourhoods^(14)^. Several studies have indicated that greater availability of supermarkets and establishments specialising in the sale of fruits and vegetables can contribute to the consumption of healthy foods^(15–18)^. On the other hand, greater availability of snack bars, fast food and convenience stores facilitates access to food with lower nutritional quality, particularly ultra-processed foods^(16–21)^.

When food stores close and are replaced by other establishments with the same marketing profile, residents are chronically exposed to certain types of food, whether healthy or not. On the other hand, variations that occur in the emergence of different types of food stores modify the characteristics of the food environment, which can promote changes in food consumption and, consequently, in the health of individuals^(22)^.

It is important to emphasise that the characteristics of the community food environment and the socio-economic context influence an individual’s diet as they constitute opportunities and/or barriers that impact the supply and consumption of healthy or unhealthy foods^(5,7,23)^. Understanding the dynamics of this environment can contribute to the formulation and implementation of health policies that reduce the inequalities that exist among communities over time, particularly in relation to access to and availability of food^(24)^. Moreover, research on the community food environment is an important aspect of building healthier food environments.

In the Latin American context, most studies are focused on different dimensions of the food environment and their impacts on diet, obesity and chronic diseases. The main works analyse food retail/provision, but there is still a gap in knowledge about the longitudinal evaluation of this dimension^(25)^.

In Brazil, some studies have assessed the retail food environment of different cities^(26–28)^, but to date, no study has focused on changes in the characteristics of this environment over time. Our objective was to assess the change in the retail food environment profile in a Brazilian metropolis over a 10-year period. The specific research questions were as follows: (1) Did the distribution of food stores in the city bring about changes in the profile of the food environment over the study period? (2) How does the profile of food stores differ by time period? (3) How are food establishments distributed throughout the city? And (4) Does the profile of food establishments differ by population density and neighbourhood income?

## Methods

### Scenario and study design

This ecological study was carried out in the city of Belo Horizonte, Minas Gerais, using secondary data obtained from a government database. Belo Horizonte is the sixth largest city in Brazil and the eighth largest in Latin America, with an estimated population of 2 512 070 inhabitants and 486 neighbourhoods grouped into 9 administrative regions^(29)^.

### Data collection

The database was developed using information on formal food establishments in Belo Horizonte registered to operate in any one or more of those years (2008, 2011, 2015 and 2018), for a total evaluation period covering 10 years. The analysed period and its time interval were determined considering the potential for evaluating the data over the period of a decade. The establishments’ addresses and main activity codes were collected according to the National Classification of Economic Activities (CNAE)^(30)^. The CNAE is an instrument to support the national standardisation of economic activity codes and classification criteria used by the various bodies of the Brazilian Tax Administration (IBGE) and organises establishments into different types according to their main activity (Supplementary material).

### Characterisation of the profile of a neighbourhood’s food environment

Based on the evaluation of each establishment, they were classified into categories. The CNAE description was used to classify these establishments according to the main activity and the predominantly commercialised foods, considering the extent and purpose of food processing and its classification as proposed by NOVA^(10)^, which categorises foods into: (a) fresh and/or minimally processed foods; (b) culinary ingredients; (c) processed foods and (d) ultra-processed foods.

Finally, establishments were aggregated into three categories, considering the percentage of food purchases for each type of establishment^(31)^. Based on this, food outlets were classified as healthy, mixed and unhealthy (Table 1). All establishments whose main activity was to provide ready-to-eat or frozen meals providing for home consumption were referred to as delivery in the present study and classified as unhealthy. This classification is based on a survey conducted in Belo Horizonte (MG) that identified a high availability of unhealthy meals in food delivery apps in the city^(32)^.

**Table 1:** Food retailer classification based on CAISAN

Types of food outlets	Food retailers	Classification
Establishments mostly selling fresh or minimally processed food	Fresh product store, butcher shop and fish market	Healthy
Mixed establishments	Bakery, restaurant, supermarket, hypermarket and dairy products	Mixed
Establishments mostly selling ultra-processed food	Minimarket, pub or bar, candy shop, beverage distributor, snack bar, convenience store, delicatessen, street food vendors and delivery*	Unhealthy

* Establishments that provide ready-to-eat or frozen meals for consumption at home, such as pizzerias and restaurants^(30–32)^.

Source: Adapted from CAISAN, 2018^(30)^.

### Geospatial data

The geographic coordinates (latitude and longitude) of the food outlets were obtained from the addresses registered with the CNAE using the Google Maps online search service (https://www.google.com.br/maps?hl=pt-BR). Data were collected in the WGS 84 Geographical Coordinate System configuration and later transformed into the Projected Coordinate System, Universal Transverse Mercator System (UTM), spindle 23S, and datum SIRGAS 2000, using the QGis 2.14.4 software.

### Sociodemographic data

Sociodemographic data included population size and total income by neighbourhood (sum of the income of all assessed households in the area) in addition to the population estimate by the year of study evaluation. The density of food outlets was also analysed according to the per capita income of the neighbourhoods, calculated as the total income of the neighbourhood divided by the number of residents in the neighbourhood.

Precise and estimated data were extracted from the 2010 Demographic Census^(29)^ and from the website of the Brazilian Institute of Geography and Statistics (https://www.ibge.gov.br/pt/inicio.html).

### Data analysis

Based on the adopted grouping, each neighbourhood’s food environment was evaluated based on income categories and seven indicators^(33)^, three to assess relative density (proportion of establishments), three to assess density per population and one to assess the disadvantage of the availability of unhealthy establishments in relation to the availability of healthy establishments in the municipality (Table 2).

**Table 2:** Food environment assessment indicators, execution formula and indicator objective

Code	Indicator	Formula	Objective
I1	Proportion of establishments that predominantly market fresh and/or minimally processed foods	nA/(nA+nB+nC)	Describe the percentage of establishments that sell fresh and/or minimally processed foods
I2	Proportion of mixed standard establishments	nB/(nA+nB+nC)	Describe the percentage of mixed standard establishments
I3	Proportion of establishments that predominantly market ultra-processed foods	nC/(nA+nB+nC)	Describe the percentage of establishments that sell ultra-processed foods
I4	Density of establishments that predominantly market fresh and/or minimally processed foods (A) per 5000 residents	(nA/estimated total population) *5000	Evaluate the availability of predominantly fresh and/or minimally processed foods
I5	Density of mixed standard establishments (B) per 5000 residents	(nB/estimated total population) *5000	Evaluate the availability of establishments that sell food in general
I6	Density of establishments that predominantly sell ultra-processed foods (C) per 5000 residents	(nC/estimated total population) *5000	Evaluate the availability of establishments that predominantly market ultra-processed foods
I7	Ratio of densities of establishments that predominantly market ultra-processed foods	I6/I4	Expresses the (dis)advantage of the availability of establishments that predominantly commercialise fresh and/or minimally processed foods with establishments that predominantly commercialise ultra-processed foods

nA = Number of establishments that predominantly market fresh and/or minimally processed foods; nB = number of mixed standard establishments; nC = number of establishments that predominantly market ultra-processed foods.

Source: Adapted from Castro Junior, 2018.

The analysis of the indicators for evaluating the food environment included the neighbourhoods (*n* 486), the average population per neighbourhood (Mean = 5000 residents) and the estimated population of the municipality per year of assessment as units of analysis^(29)^. An average population of 5000 residents was adopted, as this is the approximate value of the average population per neighbourhood. The estimated average population was used to assess the density of establishments by neighbourhood.

The density of food outlets was also analysed according to the per capita income of the neighbourhoods, calculated as the total income of the neighborhood divided by the number of residents in the neighbourhood. The 10th and 90th percentiles were considered, corresponding to the lowest and highest income neighbourhoods, respectively (*n* 101). Density results are presented in the form of tables.

To characterise the food environment of the neighbourhood in the four periods analysed, bivariate analyses were performed, including frequency, mean and standard deviation. The variation in the number of food outlets among the analysed periods (absolute and percentage) and between the final (2018) and initial (2008) periods was quantified. Generalised linear models were used to analyse the trend in the number of food outlets. The level of significance was set at *P* < 0·05. These analyses were performed using the statistical software Statistical Software for Professionals (STATA), version 14.2.

The analysis of the spatial distribution was performed using kernel maps for each category of establishments per area (km^2^) during the analysed periods. Kernel analysis allows for the detection of clusters based on the distribution pattern of points, generating a density surface of the areas with the highest occurrence of an event. Spatial analyses were performed using the QGis 2.14.4 software.

## Results

A total of 83 752 commercial food establishments were identified in the city of Belo Horizonte, with 14 540 in 2008, 17 872 in 2011, 21 274 in 2015 and 30 066 in 2018. In general, during the 10-year period, all food establishment types evaluated showed a significant increase (*P* < 0·05) in the municipality, except for hypermarkets, for which there was a reduction (*P* < 0·001), and supermarkets (*P* = 0·738), grocery stores (*P* = 0·147), and butchers (*P* = 0·316), for which there was no significant variation. Convenience stores (157·5 %; *P* < 0·001), bars (571·1 %; *P* < 0·001), street vendors (1304·4 %; *P* < 0·001) and delivery services (7088·5 %; *P* < 0·001) showed the greatest variation (Table 3).

**Table 3:** Distribution of food outlets in Belo Horizonte (MG) according to the National Classification of Economic Activities (CNAE) and year of assessment, 2008 to 2018

Category	2008		2011		2015		2018		Variation between 2008 and 2018	*P* value*
	*n*	%	*n*	%	*n*	%	*n*	%	%	
Hipermarkets	112	0·8	51	0·3	21	0·1	26	0·1	−76·8	<0·001
Supermarkets	332	2·3	282	1·6	163	0·8	389	1·3	17·2	0·738
Grocery stores	1827	12·6	1852	10·4	2027	9·5	1852	6·2	1·4	0·147
Bakery	909	6·3	681	3·8	1109	5·2	1228	4·1	35·1	<0·001
Dairy products	365	2·5	432	2·4	565	2·7	569	1·9	55·9	<0·001
Candy shops	461	3·2	502	2·8	605	2·8	509	1·7	10·4	<0·008
Butcher shop	971	6·7	894	5·0	907	4·3	919	3·1	−5·4	0·316
Fish market	38	0·3	60	0·3	87	0·4	117	0·4	207·9	<0·001
Beverage distributors	891	6·1	1041	5·8	206	0·9	1849	6·2	107·5	<0·001
Fresh product store	563	3·9	697	3·9	992	4·7	1031	3·4	83·1	<0·001
Convenience stores	776	5·3	1031	5·8	473	2·3	1998	6·7	157·5	<0·001
Restaurants	3111	21·4	3566	19·9	4467	21·0	5093	16·9	63·7	<0·001
Pubs and bars	557	3·8	2081	11·6	3708	17·4	3738	12·4	571·1	<0·001
Snack bars	3462	23·8	3455	19·3	5474	25·7	5423	18·0	56·6	<0·001
Street food vendors	113	0·8	534	3·0	111	0·5	1587	5·3	1304·4	<0·001
Delivery	52	0·4	713	4·0	359	1·7	3738	12·4	7088·5	<0·001
All establishments	14 540	100·0	17 872	100·0	21 274	100·0	30 066	100·0	106·8	<0·001

* *P*-value calculated by the generalised linear models; *P*-value < 0·05.

It can be seen that healthy establishments represented about 11·0 % of food purchase locations in 2008. In 2018, they represented approximately 7·0 %. Despite the percentage reduction, there was an increase in the number of these establishments (1572 to 2067) over the 10-year period. Mixed establishments, in turn, presented a percentage reduction over the same period from 33·2 % to 24·3 %; on the other hand, the number of establishments in this category grew over the years (4829 to 7305). The number of establishments that were considered unhealthy increased from 56·0 % in 2008 to 68·8 % in 2018, a variation of 154·3 % over the analysed period (Table 4).

**Table 4:** Distribution of food outlets in Belo Horizonte (MG) according to the predominance of commercialised foods, 2008 to 2018

Category	2008		2011		2015		2018		Variation
	*n*	%	*n*	%	*n*	%	*n*	%	%
Healthy	1572	10·8	1651	9·2	1986	9·0	2067	6·9	31·5
Mixed	4829	33·2	5012	28·0	6325	30·0	7305	24·3	51·3
Unhealthy	8139	56·0	11 209	62·7	12 963	61·0	20 694	68·8	154·3
All establishments	14 540	100·0	17 872	100·0	21 274	100·0	30 066	100·0	106·8

**Fig. 1:**
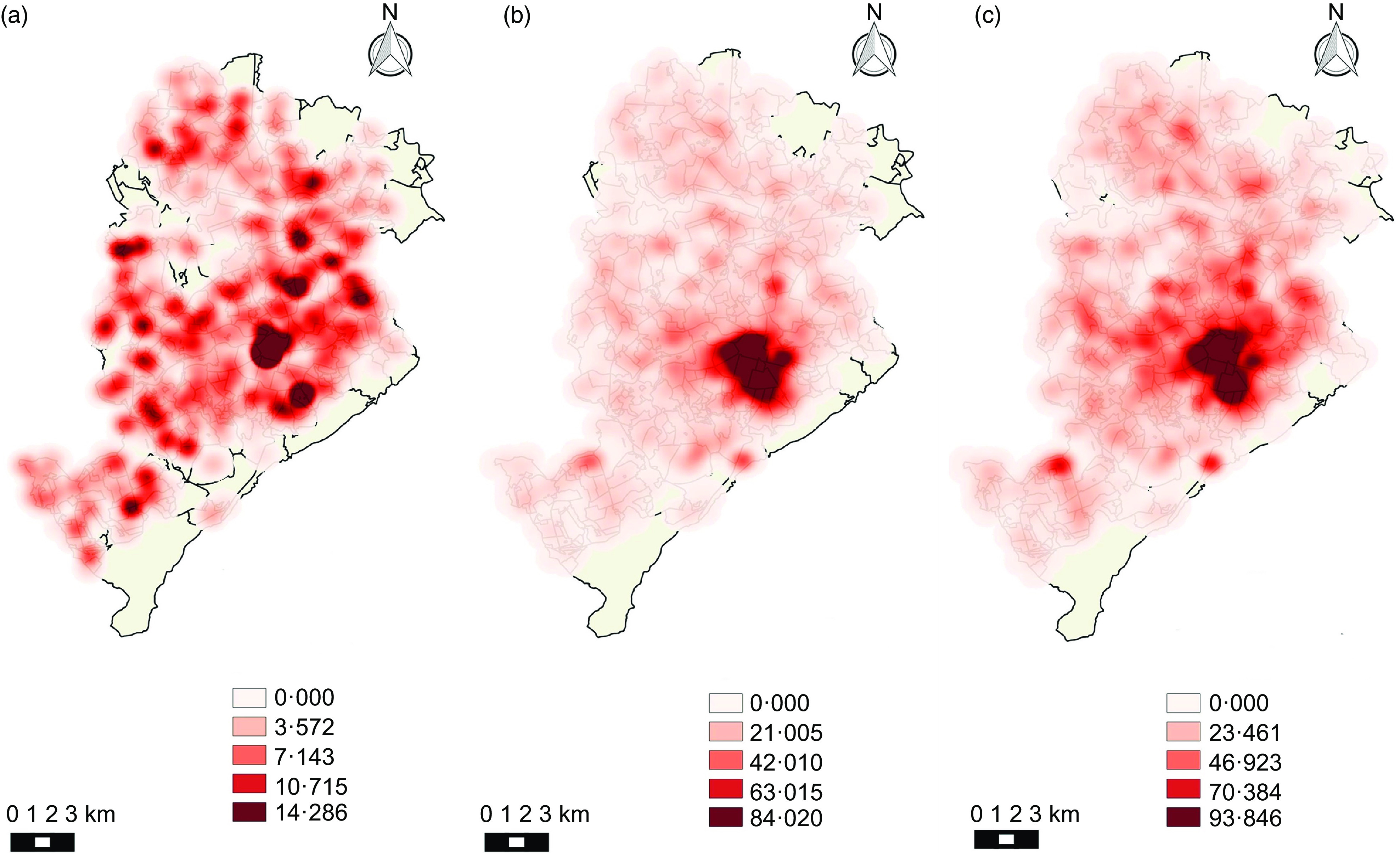
Density of healthy, mixed and unhealthy food establishments per area (km^2^) in Belo Horizonte in 2008. (a) Density of healthy establishments; (b) density of mixed establishments; (c) density of unhealthy establishments

**Fig. 2:**
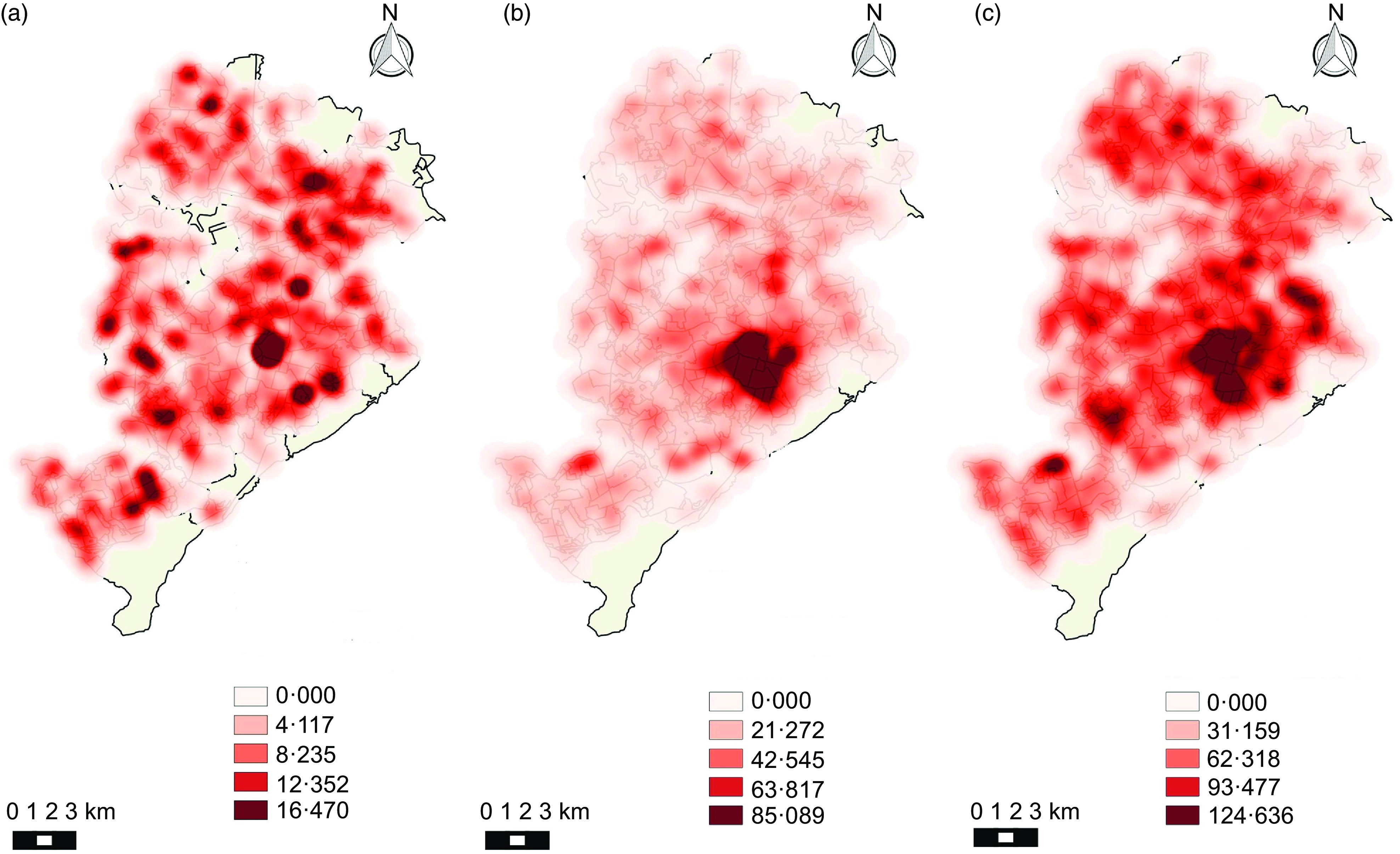
Density of healthy, mixed and unhealthy food establishments per area (km^2^) in Belo Horizonte in 2018. (a) Density of healthy establishments; (b) density of mixed establishments; (c) density of unhealthy establishments

Kernel maps show the spatial distribution of the categories of establishments during the analysed period. Figures 1 and 2 (a, b, c) show that establishments that were considered healthy did not present a pattern of agglomeration. On the other hand, mixed and unhealthy establishments presented a pattern of agglomeration in the central region of the city.

The density indicator of establishments in the municipality’s districts shows a general increase in the average density for all categories. The greatest increase in the average number of unhealthy establishments stood out, increasing from about 16 points of sale per neighborhood in 2008 (sd = 43·2) to about 42 points per neighbourhood in 2018 (sd = 68·0) (Table 5).

**Table 5:** Density of food outlets according to neighbourhood in the city of Belo Horizonte 2008 to 2018 (*n* 486)

Category	2008		2011		2015		2018	
	Mean	sd	Mean	sd	Mean	sd	Mean	sd
Healthy	3·2	7·5	3·4	6·6	4·1	6·8	4·3	7·3
Mixed	3·5	6·9	10·1	28·0	13·0	28·2	14·9	30·7
Unhealthy	16·5	43·2	22·6	52·6	26·4	54·0	42·1	68·0

From 2008 to 2018, the average size of the population residing in the municipality increased slightly (3 %), while the density of food establishments continued to increase. In 2008, there were about 3 healthy establishments for every 5000 inhabitants per district in the municipality. In 2018, there were about four establishments for the same average population of residents. On the other hand, the density of unhealthy establishments more than doubled during the same period, going from about seventeen establishments to forty-one over a 10-year period, considering the same population average per neighbourhood.

The analysis of the density ratio indicator suggests a disadvantage in the availability of healthy establishments in relation to the availability of unhealthy establishments in the municipality, with this difference accentuated over the years. In 2008, there were five unhealthy establishments for every one healthy establishment. In 2018, there were ten unhealthy establishments for every one healthy establishment.

When analysing whether the density of commercial food establishments in the city differed between the neighbourhoods at the extremes of income level, it was observed that residents of neighbourhoods where the per capita income was less than $89·27 (value corresponding to the 10th percentile) had no access to establishments that sold healthy foods, while residents of neighbourhoods where the per capita income was greater than $630·43 (value corresponding to the 90th percentile) had access to approximately seven establishments in the same category. Similarly, in higher-income neighbourhoods, there was a greater density of establishments with mixed and unhealthy commercialisation patterns, illustrating the inequalities of access to commercial food establishments according to income (Table 6).

**Table 6:** Average density of food outlets according to neighbourhood in the city of Belo Horizonte and per capita income percentiles, 2008 to 2018 (*n* 101)

Category	2008				2011				2015				2018			
	10° percentile*		90° percentile**		10° percentile*		90° percentile**		10° percentile*		90° percentile**		10° percentile*		90° percentile**	
	Mean	sd	Mean	sd	Mean	sd	Mean	sd	Mean	sd	Mean	sd	Mean	sd	Mean	sd
Healthy	0·2	0·1	7·4	2·6	0·3	0 2	6·0	2·0	0·8	0·3	6·0	2·0	0·7	0·2	6·6	2·1
Mixed	0·2	0·1	8·4	2·1	0·5	0 2	42·5	10·0	1·0	0·3	44·1	9·4	1·9	0·4	49·3	10·0
Unhealthy	0·5	0·2	55·0	15·2	2·0	0 7	63·4	18·4	3·2	1·0	65·0	18·4	7·8	2·3	83·0	21·0

* $0.00–$89.27.

** $630.43 or more.

## Discussion

In absolute and relative terms, there was an increase in all types of establishments during the study period, with the exception of hypermarkets, where there was a reduction. For establishments such as supermarkets, grocery stores, sweets shops and butchers, there was stability in the number of establishments. In general, the main increase observed refers to establishments that predominantly sell unhealthy foods (154 %), followed by mixed (51 %) and healthy foods (32 %), demonstrating that the retail food environment among formal commerce in Belo Horizonte’s food sector has changed significantly over the years, primarily in relation to the restricted availability of establishments that predominantly sell healthy foods.

The results of the present study showed a trend towards a higher density of unhealthy food establishments. There was greater growth in these establishments between 2015 and 2018, with an increase of about 60 % in the number of establishments during this period. There was greater growth in bars (571 %), street vendors (1304 %) and food delivery (7089 %). In this study, delivery refers to places that provide ready-to-eat or frozen meals for consumption at home, such as pizzerias, snack bars and restaurants. In Brazil, part of this group of establishments is characterised by providing ultra-processed food quickly and at discounted prices, similar to fast-food outlets in other countries, where the increase in this category is also notable^(32,34,35)^. It is noteworthy that Belo Horizonte is known as the ‘bar capital’, and these establishments are part of the city’s food culture, moving even the gastronomic tourism sector; this can influence and justify the growth of this type of trade^(36)^.

The increasing density of unhealthy establishments found in this study is also consistent with surveys conducted in high-income countries. In a study carried out in New York^(24)^, the average number of unhealthy establishments in the census tracts increased from three in 1990 to about six in 2010, with an emphasis on the increase in grocery stores, convenience stores, fast-food restaurants and bakeries. The increase in the density of establishments also occurred with an increase in income and population density in the census tracts evaluated.

Similarly, the results of the present study indicate that the density of commercial food establishments in Belo Horizonte is related to population growth in the municipality, albeit disproportionately. The results show that, considering the same average population size in all periods, the density of unhealthy establishments more than doubled, with a greater availability of these foods being maintained. Over a period of 10 years, the population in Belo Horizonte grew by 2·7 %. The total number of food establishments grew by 107 %.

In a similar, researchers from Melbourne, Australia, found that the density of food establishments grew at a greater and more advanced proportional rate than the residential population, particularly the density of those classified as unhealthy. As observed in Belo Horizonte, the proportion of unhealthy establishments in relation to healthy establishments was higher, indicating a retail food environment with unfavourable characteristics for healthy eating^(22)^.

The results of the present study also indicate that, regardless of the per capita income range, the density of establishments that predominantly sell unhealthy foods is considerably higher than the density of establishments that sell healthy foods. In this sense, there is evidence of inequalities in relation to the availability of food in lower-income neighbourhoods, where residents have proportionally greater availability of establishments that sell unhealthy foods, with this difference being accentuated over the period analysed.

Also considering the availability of establishments, the spatial analysis maps show a pattern of agglomeration of mixed and unhealthy establishments in the central area of the city. Another study conducted in the same location also observed a tendency for food establishments to cluster in this region, characterised by having greater purchasing power. These results indicate the importance of public policies that seek to promote food supply to prioritise the most vulnerable areas, where access and availability of food becomes more difficult, especially healthy ones^(37)^.

Thus far, few studies have sought to assess disparities in access linked to the food environment over time, especially in low- and middle-income countries^(22)^. To date, no Brazilian or international study has analysed the density, type or proportion of food outlets in a Brazilian city over time. In addition, the use of spatial analysis resources associated with statistical analysis allowed us to assess changes in the food environment in a longitudinal way, providing a more comprehensive understanding of the availability of food in the assessed city. Moreover, by including several categories of establishments that sell food, as opposed to evaluating only large establishments, the present study provides information on the trend of food commercialisation, especially for places similar to the one studied.

Another differential is the analysis of establishments categorised as delivery. Currently, there is a dynamic relationship among the dimensions of the food environment through Internet access and exposure to a wide variety of foods. The main attraction for the use of delivery services and their applications is based on practicality and convenience, allowing you to purchase food without leaving home. However, these characteristics increase individuals’ exposure to marketing strategies that encourage the consumption of ultra-processed foods, which correspond to most foods offered by this type of service^(32,38)^. As noted, the density of these establishments in Belo Horizonte grew substantially over the 10-year period, which may favour the availability of unhealthy foods.

Still on the increase of delivery establishments and due to the data used, we point out that it is not possible to evaluate the difference in the type of food offered over the period in this category, but it is important to consider the increasing availability of ultra-processed foods in various types of establishments and locations^(16–18)^, as well as the current food system in which these foods are more widely produced and marketed^(39)^. Given this, we believe that changes in the food supplied and also in the profile of these establishments may have occurred, but further studies are needed to confirm this hypothesis, especially those that seek to evaluate the consumer’s food environment, where other food characteristics such as quality, variety and price are analysed, and also of the digital food environment.

The main limitation of this study is the secondary data used in the evaluation. In some categories of establishments, such as street vendors, the variation in the absolute number between the years of assessment is not linear, with a considerable drop in 2015 compared to 2011 (from 534 to 11) and a subsequent increase in 2018 (to 1587). It is believed that this variation is due to the fact that existing establishments may not be registered and establishments that existed may have ended their activities without the regulatory bodies being aware, highlighting a possible effect of the economic crisis. Furthermore, with this type of data, it is not possible to assess the informal food trade, which is characteristic of low- and middle-income countries such as Brazil.

This study provides evidence of the disproportionate growth in the sale of unhealthy foods relative to the sale of healthy foods in a Brazilian metropolis, generating changes in the profile of the food environment. Moreover, the distribution of categories of establishments across the city is not homogeneous, especially in low-income areas, which are more exposed to unhealthy foods. Understanding the food and environmental context in which individuals are inserted is relevant to the development of long-term processes that provide better food choices. From this perspective, public nutrition policies that aim to promote availability and access to places that offer healthy foods, such as subsidies for stores selling fruits and vegetables, are important for the promotion of healthier eating environments, particularly in neighbourhoods of lower socio-economic status.

## Supplementary material

For supplementary material accompanying this paper visit https://doi.org/10.1017/S1368980022000787. click here to view supplementary material
