# Supplementary material for: Retail food environment in a Brazilian metropolis over the course of a decade: evidence of restricted availability of healthy foods
Source: Public Health Nutr. 2022 Mar 28;25(9):2584–92. doi: 10.1017/S1368980022000787 (PMC9991744; doi:10.1017/S1368980022000787)
Supplement: Supplementary file 1 [file S1368980022000787sup001.docx]

**Supplementary material** – Description of food sales establishments evaluated according to their respective National Classification of Economic Activities (CNAE)

| **CNAE** | **Description CNAE** | **Explanatory** |
| --- | --- | --- |
| 4711301 | Retail sale of general merchandise, with a predominance of food products - hypermarkets | Activities of commercial establishments with predominant sale of varied food products and that also offer a wide range of other goods, such as household utensils, cleaning and personal care products, clothing, hardware, etc. with a sales area exceeding 5000 square meters |
| 4711302 | Retail sale of general merchandise, with a predominance of food products - supermarkets | The activities of commercial establishments with predominant sale of varied food products and that also offer a wide range of other goods, such as household utensils, cleaning and personal care products, clothing, hardware, etc., with a sales area between 300 to 5000 square meters |
| 4712100 | Retail sales of general merchandise, with a predominance of food products - mini-markets, grocery stores, and warehouses | Activities of commercial establishments with and without self-service and with predominant sale of various food products in mini-markets, grocery stores, warehouses, emporiums, dry and wet, with a sales area of less than 300 square meters |
| 4721102 | Bakery and confectionery with a predominance of resale | Retail trade of bread and doughnuts, cakes, pies, and other bakery products when the resale of other products is predominant |
| 4721103 | Retail trade of dairy and cold products | Retail trade of milk and dairy products, such as butter, cream, yogurt, and curds; cold cuts and preserved meats; and preserved fruits, vegetables, and similar |
| 4721104 | Retail trade of sweets, candies, bonbons, and similar | Retail trade of sweets, candies, bonbons, and similar |
| 4722901 | Retail trade of meat - butchers | Retail trade of fresh, refrigerated, and frozen beef, pork, goat, sheep, and equine meat; fresh, frozen, or refrigerated slaughtered poultry; small animals slaughtered; rabbits, ducks, turkeys, chickens, and the like; and for the slaughter of animals associated with the trade |
| 4722902 | Fish market | Retail trade of fresh, frozen, preserved, or refrigerated fish, crustaceans, and mollusks |
| 4723700 | Beverage distributors | Retail trade of alcoholic and non-alcoholic beverages, not consumed at the point of sale |
| 4724500 | Retail trade of fresh products | Retail trade of fruits, vegetables, live birds, and other small animals for food |
| 4729699 | Retail trade of food products in general or specialized in food products not otherwise specified | Includes natural and diet products, frozen foods, honey, ground coffee, ice cream, packaged, jar, and similar stores; commercial establishments predominantly selling industrialized food products (convenience stores) in addition to other non-food products; commercial establishments selling a variety of food products (deli stores) |
| 5611201 | Restaurants and similar | Activities of selling and serving prepared food, with or without alcoholic beverages to the general public, with full service, including self-service restaurants or food by the kilo and restaurant and bar activities on vessels operated by third parties |
| 5611202 | Bars and other establishments specializing in serving drinks | Activities to serve alcoholic beverages, with or without entertainment, to the general public, with full service offering food |
| 5611203 | Snack bars, tea houses, juice bars, and similar | Food service for on-site consumption, with or without the sale of alcoholic beverages, in establishments that do not offer full service, such as snack bars, fast food, pastries, tea houses, juice bars and similar, and ice cream parlors, with on-site consumption, self-made or not |
| 5612100 | Mobile food services and self-service machines | Food service of prepared food for the general public, in open places, permanent or not, such as trailers, carts, and other types of street food prepared for immediate consumption; sale of prepared food using automatic service machines |
| 5620104 | Provision of food prepared mainly for home consumption | Preparation of cooked meals or dishes, including frozen ones, delivered or served at home |
